# Inhibition of cGAS-STING signaling pathway alleviates high glucose-induced mesothelial-mesenchymal transition in human peritoneal mesothelial cell line HMrSV5

**DOI:** 10.1007/s11626-025-01107-1

**Published:** 2025-08-28

**Authors:** Fuxing Dong, Luli Zheng, Fuyuan Hong

**Affiliations:** 1 Department of Nephrology, Fujian Provincial Hospital, Shengli Clinical Medical College of Fujian Medical University, Fuzhou University Affiliated Provincial Hospital, Fuzhou, China; 2 Department of Blood Purification, Fujian Provincial Hospital, Shengli Clinical Medical College of Fujian Medical University, Fuzhou University Affiliated Provincial Hospital, Fuzhou, China

**Keywords:** Peritoneal fibrosis, CGAS-STING signaling pathway, Mesothelial-mesenchymal transition, Inflammation

## Abstract

The mesothelial-mesenchymal transition (MMT) of peritoneal mesothelial cells is a critical factor contributing to the progression of peritoneal fibrosis. This study aimed to explore the effect of cGAS-STING signaling pathway on the MMT process in peritoneal mesothelial cells. The expressions of cGAS, STING, α-SMA, and Vimentin in HMrSV5 cells treated with high glucose were analyzed using WB. Subsequently, si-cGAS and the cGAS inhibitor RU.521 were employed to intervene in HMrSV5 cells. qPCR was utilized to evaluate the expression levels of genes involved in the cGAS-STING signaling pathway (cGAS, STING, IRF3, TBK1) and MMT-related genes (E-cadherin, Vimentin, α-SMA, TGF-β1). The protein expressions of the cGAS-STING signaling pathway and MMT-related proteins were detected by WB. The invasive capacity of cells in each cell was assessed using a Transwell assay, and the levels of pro-inflammatory cytokines (IL-6, TNF-α) in the supernatants of each cell were measured by ELISA. In the present study, we found that the expressions of cGAS, p-STING/STING, p-IRF3/IRF3, and p-TBK1/TBK1 proteins were significantly upregulated in HG-treated HMrSV5 cells. Furthermore, the activation of the cGAS-STING signaling pathway could be effectively suppressed in HMrSV5 cells transfected with si-cGAS or treated with RU.521. Additionally, treatment with si-cGAS or RU.521 not only attenuated the invasive capacity of HMrSV5 cells but also decreased the levels of pro-inflammatory cytokines and inhibited the expression of MMT-related markers. Suppression of the cGAS-STING signaling pathway mitigates HG-induced MMT in the human peritoneal mesothelial cell line HMrSV5.

**Supplementary Information:**

The online version contains supplementary material available at 10.1007/s11626-025-01107-1.

## Introduction

In China, the prevalence of chronic kidney disease (CKD) is approximately 10.8%, while the incidence of CKD among elderly individuals aged 65 and above exceeds 30% (Wang *et al*. 2023; Lin *et al*. 2024). As renal function progressively declines, CKD advances to end-stage renal disease (ESRD). For patients with ESRD, peritoneal dialysis (PD) is widely acknowledged as an effective alternative therapy (Sakurada *et al*. 2024; Flythe *et al.* 2024; Jin *et al*. 2024). Nevertheless, prolonged exposure to peritoneal dialysate may induce peritoneal fibrosis (PF), which compromises peritoneal function and ultimately leads to PD failure (Huang *et al*. 2024; Ito *et al*. 2024; Wang *et al*. 2024a, b). Consequently, preventing PF during PD treatment is crucial for ensuring the success of this therapeutic approach.

Epithelial-to-mesenchymal transition (EMT) is intricately associated with the onset and progression of fibrosis (Youssef *et al.* 2023; Perez-Moreno *et al*. 2024). EMT refers to a biological process wherein epithelial cells, under the influence of high glucose and transforming growth factor-β (TGF-β), lose their expression of epithelial-specific markers and acquire a myofibroblast phenotype (Jacobs *et al*. 2024; Liu *et al*. 2024a, b; Long *et al*. 2024). Mesothelial-mesenchymal transition (MMT) is a specific form of EMT that occurs mainly in mesothelial cells such as the pleura and peritoneum. MMT belongs to type II EMT, which is associated with pathophysiological processes such as tissue repair, wound healing, and fibrosis. The transdifferentiation of human peritoneal mesothelial cells into fibroblasts induced by high glucose is regarded as a pivotal mechanism underlying PF (Yu *et al*. 2018; Zhao *et al*. 2019; Masola *et al*. 2022). Consequently, investigating the mechanisms of MMT in peritoneal mesothelial cells and identifying effective preventive interventions hold substantial theoretical and clinical significance for enhancing the quality of life in patients with ESRD.

The cyclic GMP-AMP synthase (cGAS)–stimulator of interferon genes (STING) signaling pathway is an intracellular immune pathway that detects cytosolic DNA damage and viral infections, thereby initiating an innate immune response (Dvorkin *et al*. 2024; Liu *et al*. 2024a, b). Recent studies have demonstrated that the cGAS-STING signaling pathway plays a pivotal role in the regulation of fibrosis-associated diseases. Jiang *et al*. found that hypoxia can promote the progression of renal fibrosis in mice by activating the cGAS-STING signaling pathway (Jiang *et al*. 2023). In addition, Xie *et al*. demonstrated that fluvoxamine can attenuate the progression of bleomycin-induced pulmonary fibrosis in mice by inhibiting the cGAS-STING signaling pathway (Xie *et al*. 2023). However, it is still unclear whether the cGAS-STING signaling pathway is involved in the occurrence and development of PF. In this study, we established an in vitro high-glucose-induced MMT model of peritoneal mesothelial cells to investigate the effects of the cGAS-STING signaling pathway on MMT.

## Materials and methods

### Reagents

Antibodies against cGAS, STING, p-STING, IRF3, p-IRF3, TBK1, p-TBK1, E-cadherin, Vimentin, α-SMA, TGF-β1, Factor VIII, and β-actin were from Abcam (Cambridge, UK). RU.521 was purchased from MedChemExpress (Monmouth Junction, NJ). Human IL-6 ELISA Kit and human TNF-α ELISA Kit were purchased from Shanghai Enzyme-linked Biotechnology Co., Ltd. (Shanghai, China).

### Cell culture and treatment

Human peritoneal mesothelial cell line HMrSV5 was purchased from BeNa Culture Collection (Beijing, China) in April 20, 2020. BeNa Culture Collection provided STR detection report of HMrSV5 cells in May 16, 2025. DNA was extracted using Axygen’s genome extraction kit, amplified using the 21-STR amplification protocol, and the STR loci and the sex gene Amelogenin were detected on the ABI 3730XL genetic analyzer. The results showed that the cell was a single-origin human cell with no cross-contamination. Besides, PCR was used to detect whether HMrSV5 cells were contaminated by *Mycoplasma* on August 14, 2024, at BeNa Culture Collection. We ensure that the cells used in the experiment are not contaminated with *Mycoplasma*. HMrSV5 cells were cultured in DMEM supplemented with 15% fetal bovine serum and 1% penicillin/streptomycin at 37 °C in an atmosphere containing 5% CO_2_.

The experiment was divided into five groups: the Control group, the HG group, the RU.521 group, the si-NC group, and the si-cGAS group. The HMrSV5 cells in the HG, RU.521, si-NC, and si-cGAS groups were treated with 4.25% glucose for 48 h. In the RU.521 group, HMrSV5 cell lines were treated with 1 μM RU.521 for 48 h.

As for cell transfection, the si-cGAS-1, si-cGAS-2, si-cGAS-3, and si-NC were purchased from Hanhang Science and Technology Co., LTD (Shanghai, China). The sequences of siRNAs targeting human cGAS mRNA were: si-cGAS-1: 5′-GACGUUACAGUCUGAUGAATT-3′, si-cGAS-2: 5′-CCAUCUACGUGAUCGAGUATT-3′, and si-cGAS-3: 5′-GGAUCAUCUACUGGACUGATT-3′. Cell transfection was performed using Lipofectamine 3000 reagent. After transfection for 48 h, the following experiments were performed.

### Cell identification

The expressions of Factor VIII (Negative protein) and Vimentin (Positive protein) in HMrSV5 cells were detected by immunofluorescence to identify it. The treated cells were fixed with 4% paraformaldehyde for 30 min, permeated with 0.5% Triton X-100 for 20 min, closed at 37 °C with 5% BSA for 30 min, and incubated at 4 °C overnight with Factor VIII (1:200) and Vimentin (1:200) antibodies, respectively. After washing, the fluorescent secondary antibody was added, and the film was sealed with DAPI and observed under a fluorescence microscope.

### Western blotting (WB)

We discard the cell culture medium from the dish, add 100 μL of cell lysate per well, and incubate on ice for 20 min. We scrape the cells to one side using a cell scraper, transfer the suspension into a pre-labeled EP tube using a pipette, and centrifuge at 12,000 rpm for 10 min. We discard the pellet, carefully transfer the supernatant to a new EP tube (for BCA protein quantification), and store the total protein at − 20 °C. Protein concentration was determined using the BCA assay kit, followed by protein denaturation. Samples were then subjected to SDS-PAGE for 1.5 h, and subsequently transferred onto a PVDF membrane using a constant current of 300 mA for 1.5 h. The membrane was incubated with the primary antibody overnight at 4 °C, followed by incubation with the secondary antibody at room temperature for 2 h the next day. After washing the membrane, it was immersed in chemiluminescent substrate solution and placed in the sample chamber of an ultra-sensitive chemiluminescence imaging system for development and imaging.

### Quantitative real-time PCR (qRT-PCR)

Cells were harvested in culture dishes. The cell culture medium was aspirated, and Trizon Reagent was added to each dish at a volume of 1 mL per dish based on the number of cells. Subsequently, 0.2 mL of chloroform was added for every 1 mL of Trizon used. miRNA was extracted using an mRNA ultra-pure extraction kit. The concentration and purity (OD260/OD280) of the mRNA were measured using a UV–VIS spectrophotometer. cDNA was synthesized using an mRNA reverse transcription kit, and fluorescence quantitative PCR was performed using a fluorescent PCR instrument. The reaction system consisted of the following components: 10 μL of miRNA Universal SYBR qPCR Master Mix or 10 μL of 2 × SYBR Green PCR Master Mix, 1 μL of cDNA, 0.4 μL of upstream primer, 0.4 μL of downstream primer, and 8.2 μL of RNase-Free dH_2_O. The reaction steps were as follows: pre-denaturation at 95 °C for 10 min, denaturation at 95 °C for 10 s, annealing at 58℃ for 30 s, extension at 72 °C for 30 s, with a total of 40 cycles. β-Actin was used as the internal reference, and the relative gene expression was calculated using the 2-^△△Ct^ method. The sequences of the primers are shown in Table 1..

**Table 1.** Primer sequence

Gene symbol	Forward primer	Reverse primer
cGAS	AAGGCCTGCGCATTCAAAACT	AAGGATAGCCGCCATGTTTCTTCTT
STING	CCGGGAGGCAGAAGATGC	CAGGCACTCAGCAGAACCAA
IRF3	CACTGAAGCGGCTGTTGGT	AGGAGATGGTCTGCTGGAAG
TBK1	AGACAAAGCAGAACGTAGATTAGC	GCGTCATAGCTTTTGTGGCA
E-Cadherin	AATCTGAAAGCGGCTGATACTG	CCATTCGTTCAAGTAGTCATAGTCC
Vimentin	GGATTCACTCCCTCTGGTTG	TGATGCTGAGAAGTTTCGTTG
α-SMA	GCGATCTCACCGACTACCTG	GCCGACTCCATACCGATGAA
TGF-β1	CCGACTACTACGCCAAGGA	AACCACTGCCGCACAACTC
β-Actin	TGGCACCCAGCACAATGAA	CTAAGTCATAGTCCGCCTAGAAGCA

### Cell invasion assay

The cells were collected and re-suspended with serum-free medium. The cells were inoculated into the upper compartment of the transwell chamber covered with matrix glue, and the lower compartment was added with PBS medium. After 24-h culture in a CO_2_ incubator at 37 °C, we remove the cells, discard the medium, and stain with 0.1% crystal violet for 1 h. The cells in the chamber were wiped with a cotton swab and observed under a fluorescence microscope. After the photo was taken, the staining solution was removed, 33% acetic acid was added, and the absorbance of each hole was determined by microplate reader at 562-nm wavelength.

### Enzyme-linked immunosorbent assay (ELISA)

The IL-6 and TNF-α content assay was performed according to the manufacturer’s protocol.

### Statistical analyses

SPSS 26.0 was applied for statistical analysis, and experimental data were expressed as mean ± standard deviation (x ± s). One-Way ANOVA was used for comparison between groups, and *t*-test was selected for two-way comparison, and *P* < 0.05 was considered a statistically significant difference.

## Results

### The cGAS-STING signaling pathway was activated in high-glucose treated HMrSV5 cells

**Figure 1.**
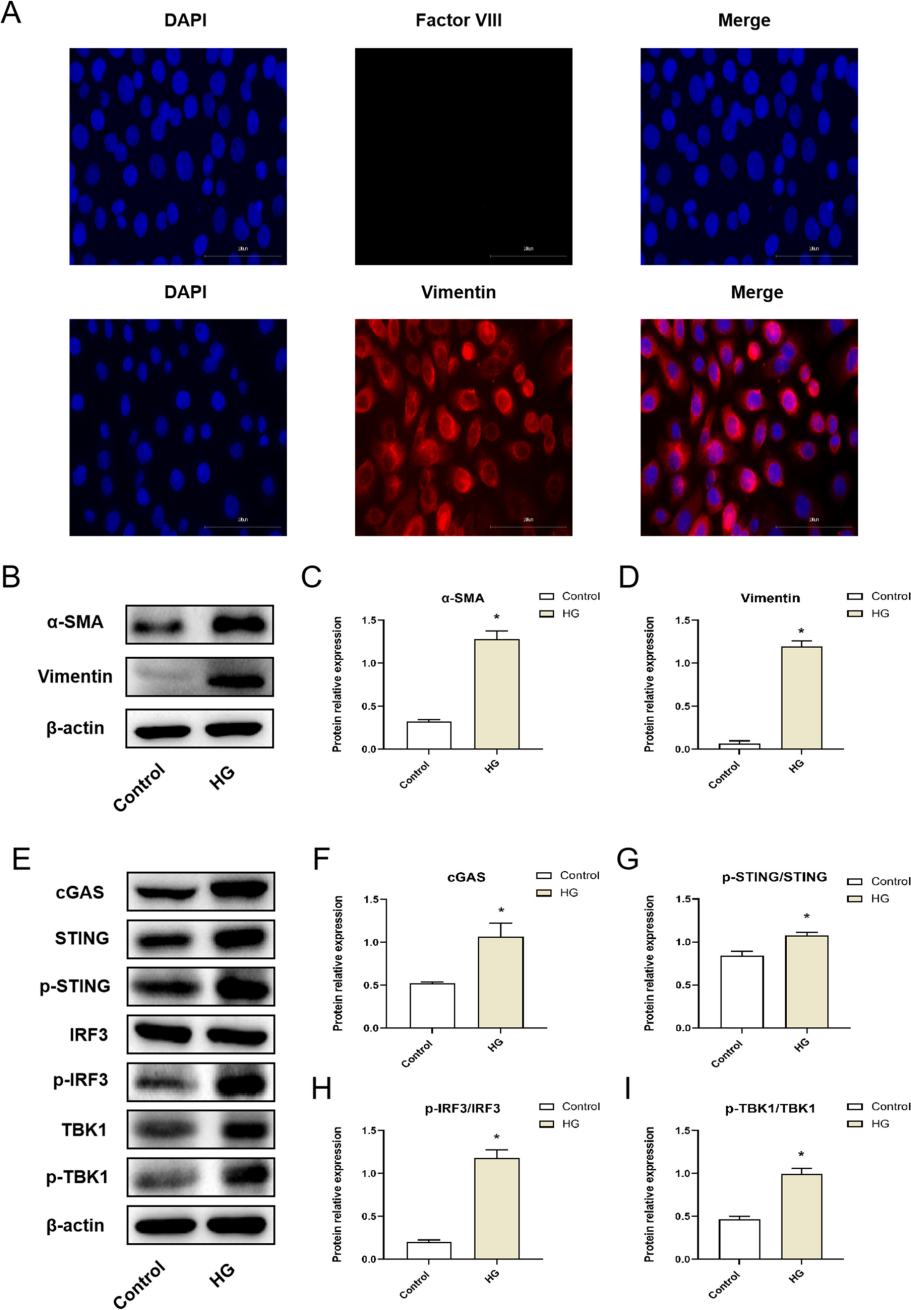
The cGAS-STING signaling pathway was activated in high-glucose treated HMrSV5 cells. (*A*) The HMrSV5 cell marker protein (Negative protein: Factor VIII, Positive protein: Vimentin) was identified by immunofluorescence. (*B*) The protein expressions of α-SMA and Vimentin in HMrSV5 cells were detected by WB. (*C*, *D*) Quantitative analysis of protein expression, (*C*) α-SMA, (*D*) Vimentin. (*E*) The protein expressions of cGAS, p-STING/STING, p-IRF3/IRF3, and p-TBK1/TBK1 in HMrSV5 cells were detected by WB. (*F***–***I*) Quantitative analysis of protein expression, (*F*) cGAS, (*G*) p-STING/STING, (*H*) p-IRF3/IRF3, (*I*) p-TBK1/TBK1. Data are presented as the mean ± SD. *P < 0.05 vs. Control.

Immunofluorescence was used to identify the marker proteins of Factor VIII and Vimentin in HMrSV5 cells (Fig. 1*A*); the results showed that Vimentin was expressed positively and Factor VIII was expressed negatively in HMrSV5 cells. After that, WB was used to detect the expression of MMT-related proteins (α-SMA, Vimentin) in HMrSV5 cells treated with high glucose for 48 h (Fig. 1*B*). The analysis results are shown in Fig. 1*C*, *D*. The α-SMA and Vimentin protein expressions of HMrSV5 cells in the HG group were significantly increased compared with the Control group (*P* < 0.05). As shown in Fig. 1*E*, the cGAS, p-STING/STING, p-IRF3/IRF3, and p-TBK1/TBK1 protein expressions of HMrSV5 cells were detected by WB, and the analysis results are shown in Fig. 1*F*–*I*. Compared with the Control group, the cGAS, p-STING/STING, p-IRF3/IRF3, and p-TBK1/TBK1 protein expressions of HMrSV5 cells in the HG group were significantly increased (*P* < 0.05).

### Effect of interfering cGAS on the mRNA and protein expression of cGAS-STING signaling pathway in HMrSV5 cells treated with high glucose

**Figure 2.**
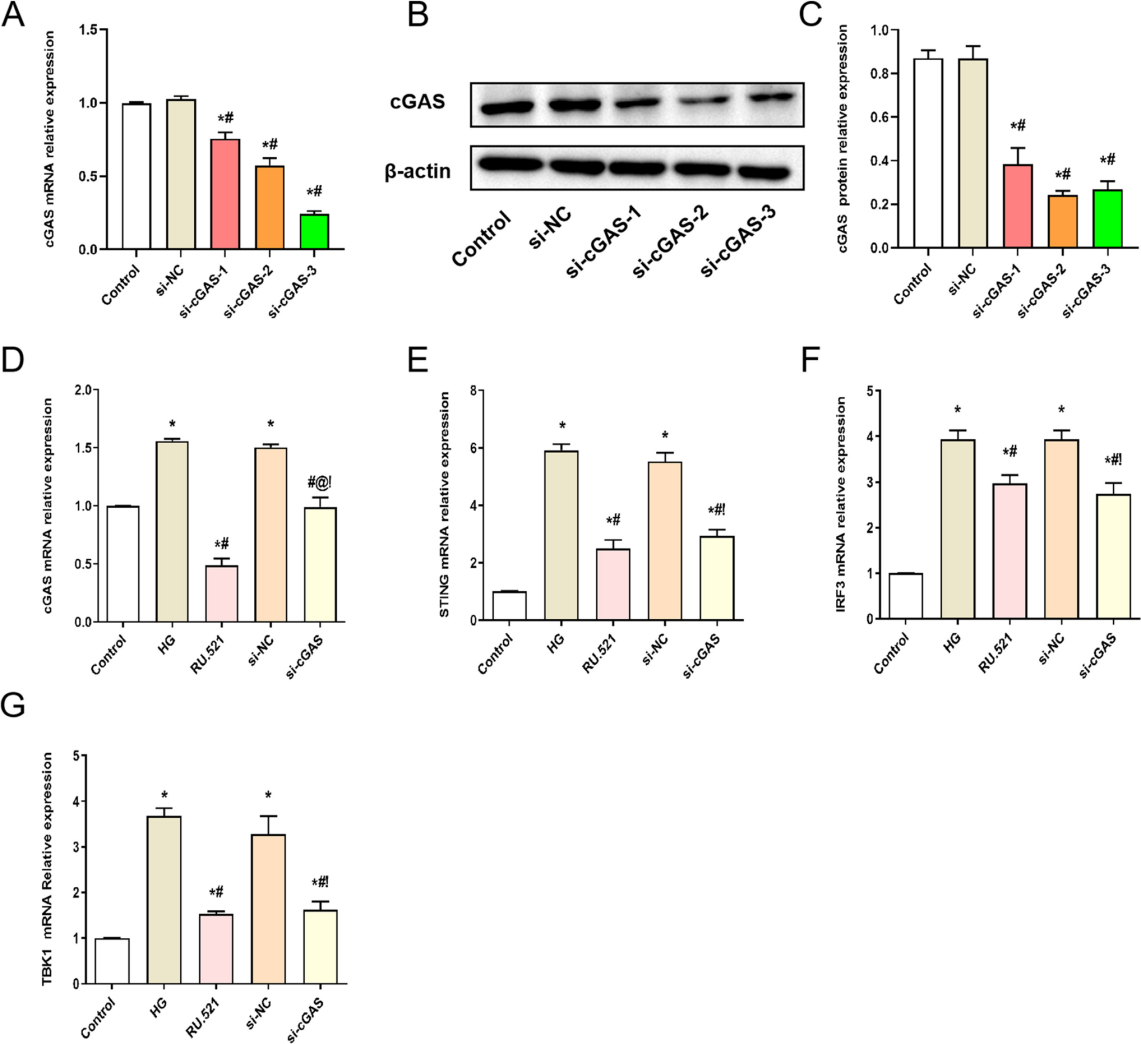
Effect of interfering cGAS on the mRNA expression of cGAS, STING, IRF3, and TBK1 in HMrSV5 cells treated with high glucose. (*A*) Transfection efficiency of si-cGASs in HMrSV5 cells was verified by qRT-PCR, and (*B*, *C*) WB, (*D*) cGAS mRNA, (*E*) STING mRNA, (***F***) IRF3 mRNA, (*G*) TBK1 mRNA were detected by qRT-PCR. Data are presented as the mean ± SD. *P < 0.05 vs. Control, #*P* < 0.05 vs. HG.

To inhibit the cGAS expression in HMrSV5 cells, we first constructed three si-cGAS and transfected them into HMrSV5 cells. The silencing effect of cGAS was verified by qPCR (Fig. 2*A*) and WB (Fig. 2*B*, *C*). Compared with the si-NC group, the mRNA and protein expressions of cGAS in si-cGAS-1, si-cGAS-2, and si-cGAS-3 groups were significantly decreased in HMrSV5 cells (*P* < 0.05). According to the results of silencing cGAS, we used si-cGAS-3 for follow-up experiments. As shown in Fig. 2*D*–*G*, the cGAS, STING, IRF3, and TBK1 mRNA expressions in HMrSV5 cells were detected by qRT-PCR. The cGAS, STING, IRF3, and TBK1 mRNA expressions of HMrSV5 cells in the HG group were significantly increased compared with the Control group (*P* < 0.05). However, compared with the HG group, the cGAS, STING, IRF3, and TBK1 mRNA expressions of HMrSV5 cells in the RU.521 group and the si-cGAS group were significantly decreased (*P* < 0.05).

### Effect of interfering cGAS on the mRNA and protein expression of cGAS-STING signaling pathway in HMrSV5 cells treated with high glucose

**Figure 3.**
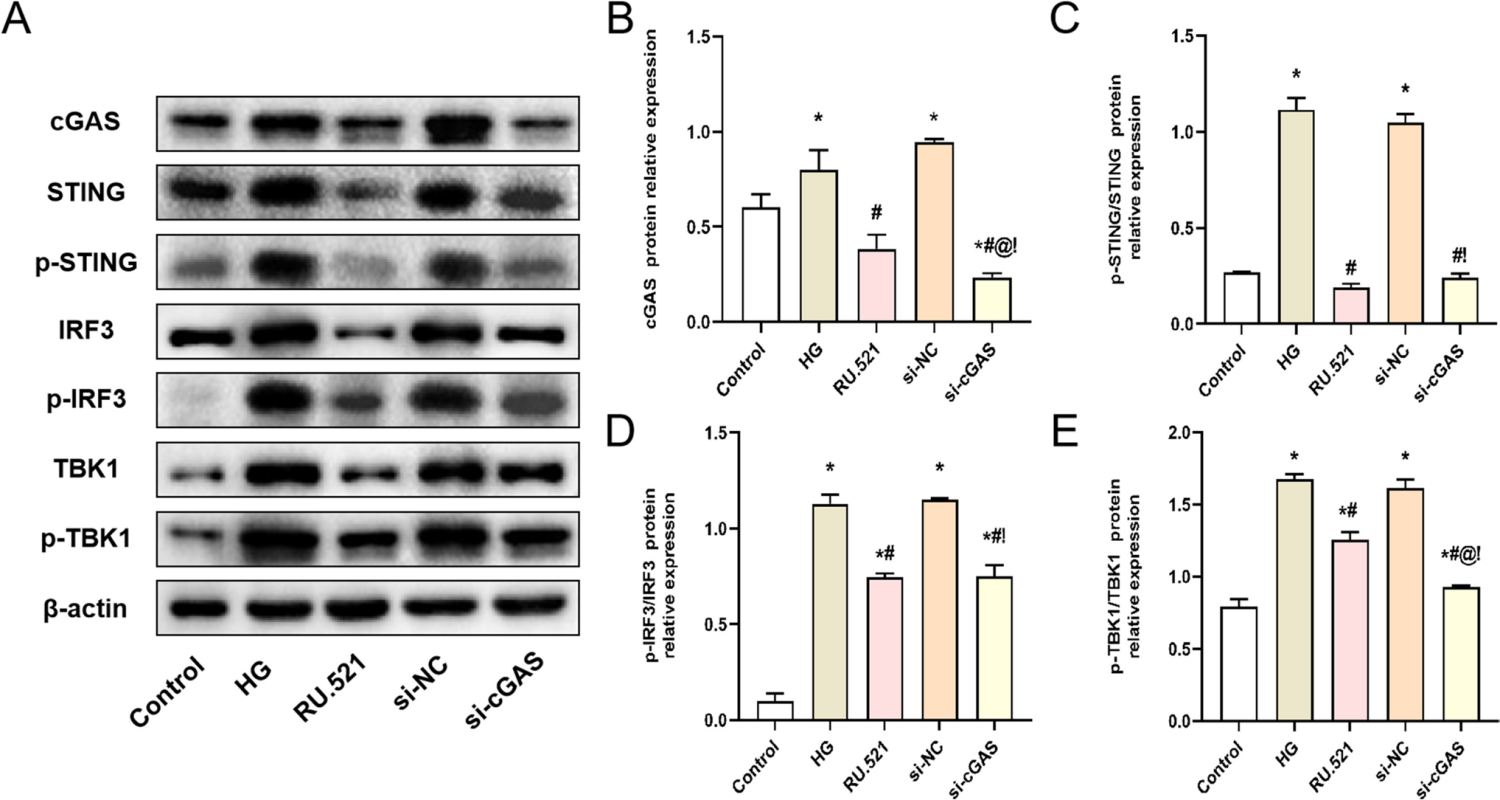
The protein expressions of cGAS, p-STING/STING, p-IRF3/IRF3, and p-TBK1/TBK1 in HMrSV5 cells were detected by WB. (*A*) The test strips of WB. (*B*–*E*) Quantitative analysis of protein expression, (*B*) cGAS, (*C*) p-STING/STING, (*D*) p-IRF3/IRF3, (*E*) p-TBK1/TBK1. Data are presented as the mean ± SD. *P < 0.05 vs. Control, #*P* < 0.05 vs. HG.

Furthermore, we investigated the effects of RU.521 and si-cGAS on the protein expression levels of the cGAS-STING signaling pathway in HMrSV5 cells through WB (Fig. 3*A*). The analysis results are shown in Fig. 3*B*–*E*. The cGAS, p-STING/STING, p-IRF3/IRF3, and p-TBK1/TBK1 protein expressions of HMrSV5 cells in the HG group were significantly increased compared with the Control group (*P* < 0.05). However, compared with the HG group, the cGAS, p-STING/STING, p-IRF3/IRF3, and p-TBK1/TBK1 protein expressions of HMrSV5 cells in the RU.521 group and the si-cGAS group were significantly decreased (*P* < 0.05).

### Effect of interfering cGAS on the cell invasion ability in HMrSV5 cells treated with high glucose

**Figure 4.**
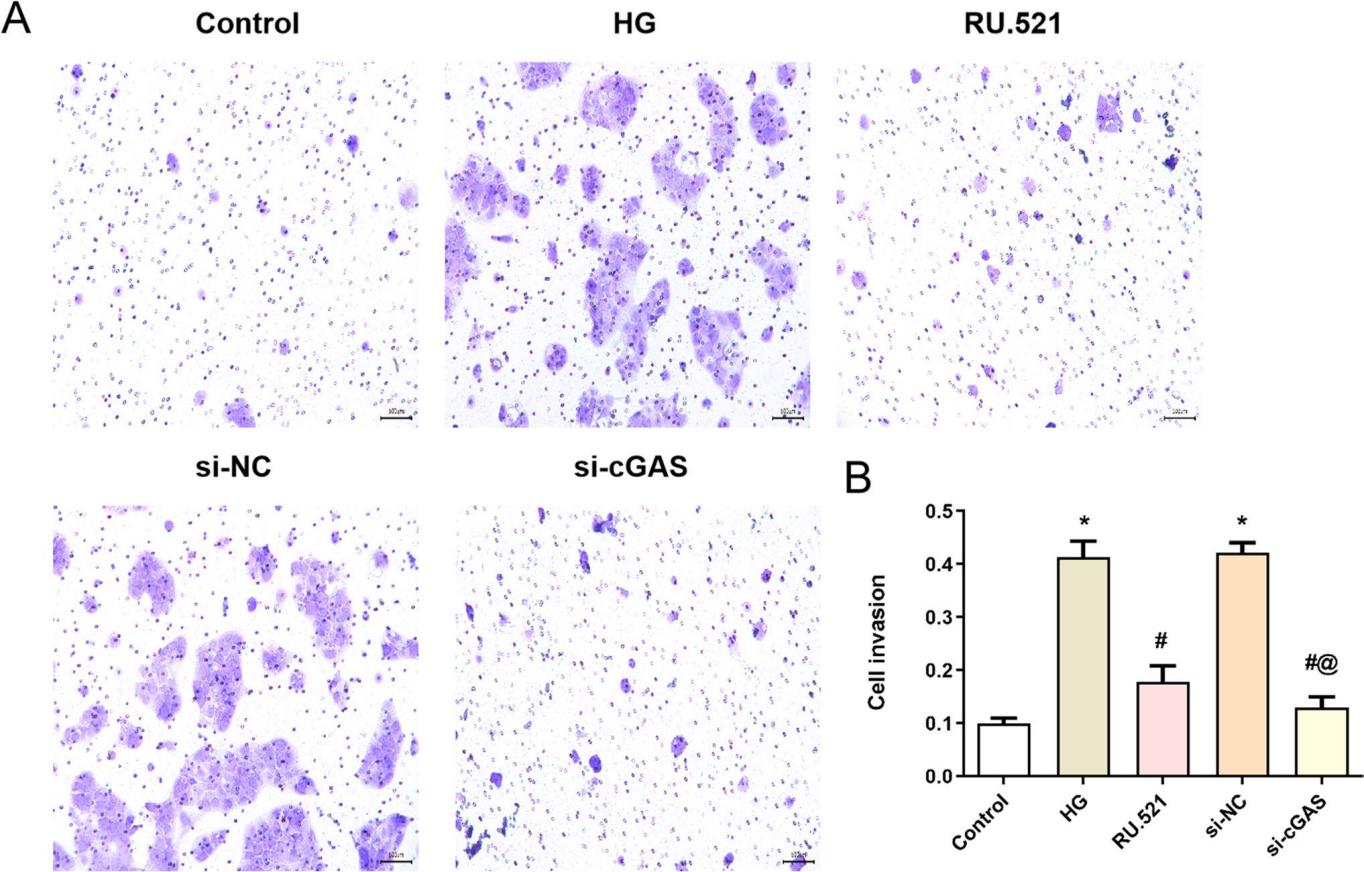
Effect of interfering cGAS on the cell invasion ability in HMrSV5 cells treated with high glucose. (*A*) The invasion of HMrSV5 cells is detected by Transwell assay. (*B*) Quantitative analysis of invasion of HMrSV5 cells. Data are presented as the mean ± SD. *P < 0.05 vs. Control, #*P* < 0.05 vs. HG.

To further investigate interfering cGAS on the invasion ability of HMrSV5 cells, the Transwell invasion assay was used to detect it (Fig. 4*A*). The results are shown in Fig. 4*B*; the invasion of HMrSV5 cells in the HG group is significantly increased compared with the Control group (*P* < 0.05). Additionally, compared with the HG group, the invasion ability of HMrSV5 cells is significantly decreased in the RU.521 group and the si-cGAS group (*P* < 0.05).

### Effect of interfering cGAS on the expression of MMT-related genes and inflammatory factors in HMrSV5 cells subjected to high glucose treatment

**Figure 5.**
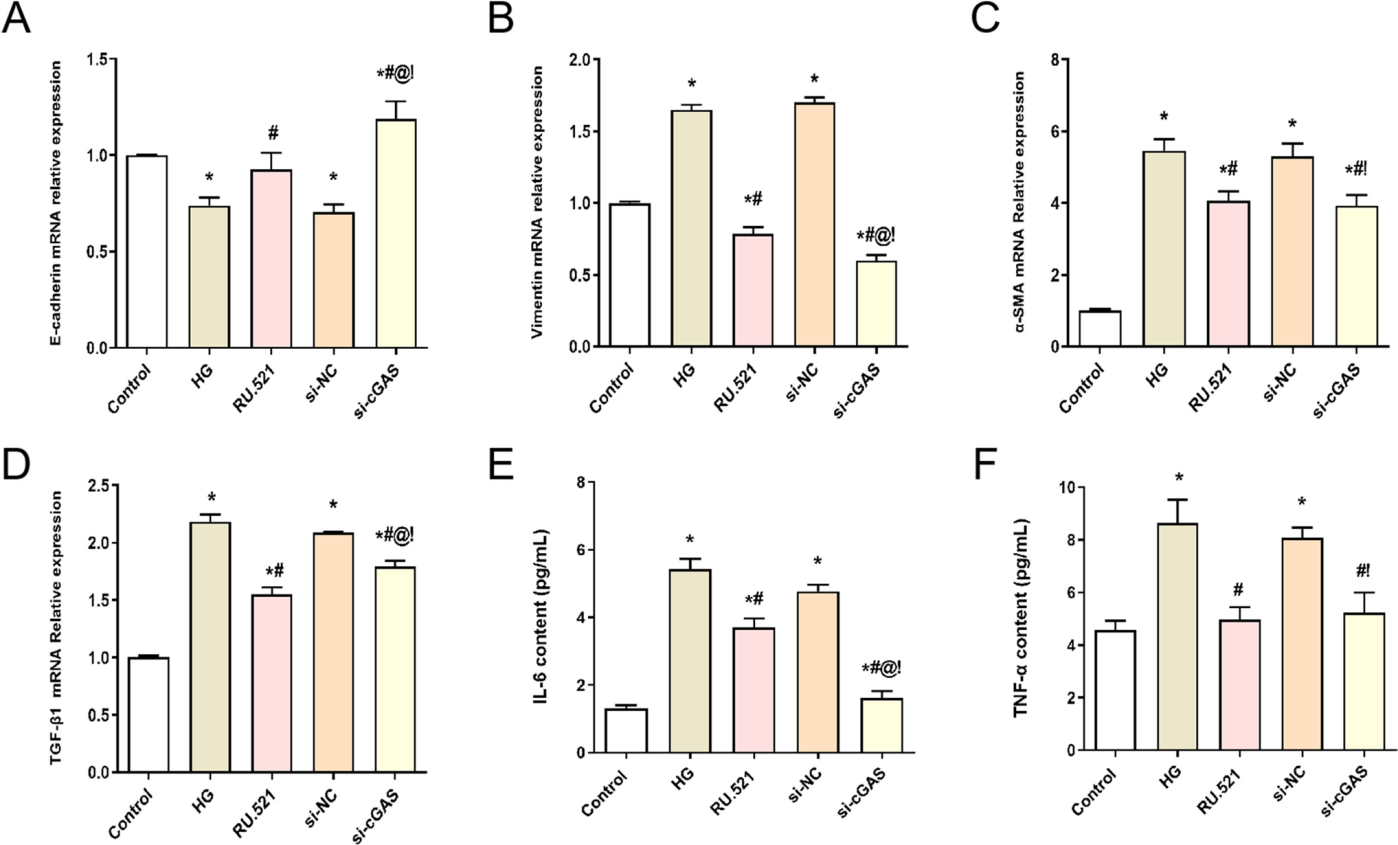
Effect of interfering cGAS on the expression of MMT-related genes and inflammatory factors in HMrSV5 cells subjected to high glucose treatment. (*A*) E-cadherin mRNA, (*B*) Vimentin mRNA, (*C*) α-SMA mRNA, and (*D*) TGF-β1 mRNA were detected by qRT-PCR. (*E*) IL-6 and (*F*) TNF-α concentrations were detected by ELISA. Data are presented as the mean ± SD. *P < 0.05 vs. Control, #*P* < 0.05 vs. HG.

As shown in Fig. 5*A*–*D*, the MMT-related gene (E-cadherin, Vimentin, α-SMA, and TGF-β1) expressions in HMrSV5 cells were detected by qRT-PCR. Compared with the Control group, the Vimentin, α-SMA, and TGF-β1 mRNA expressions of HMrSV5 cells in the HG group were significantly increased (*P* < 0.05), while the E-cadherin mRNA expression of HMrSV5 cells in the HG group was significantly decreased (*P* < 0.05). However, compared with the HG group, the Vimentin, α-SMA, and TGF-β1 mRNA expressions of HMrSV5 cells in the RU.521 group and the si-cGAS group were significantly decreased (*P* < 0.05), while the E-cadherin mRNA expression of HMrSV5 cells was significantly increased (*P* < 0.05).

As shown in Fig. 5*E*, *F*, the levels of IL-6 and TNF-α in different groups of HMrSV5 cells were detected by ELISA. Compared with the control group, the IL-6 and TNF-α concentrations in the HG group were significantly increased in HMrSV5 cells (*P* < 0.05). In addition, compared with the HG group, the IL-6 and TNF-α concentrations in the RU.521 group and the si-cGAS group were significantly decreased in HMrSV5 cells (*P* < 0.05).

### cGAS silencing alleviates the expression of MMT-related proteins induced by high glucose in HMrSV5 cells

**Figure 6.**
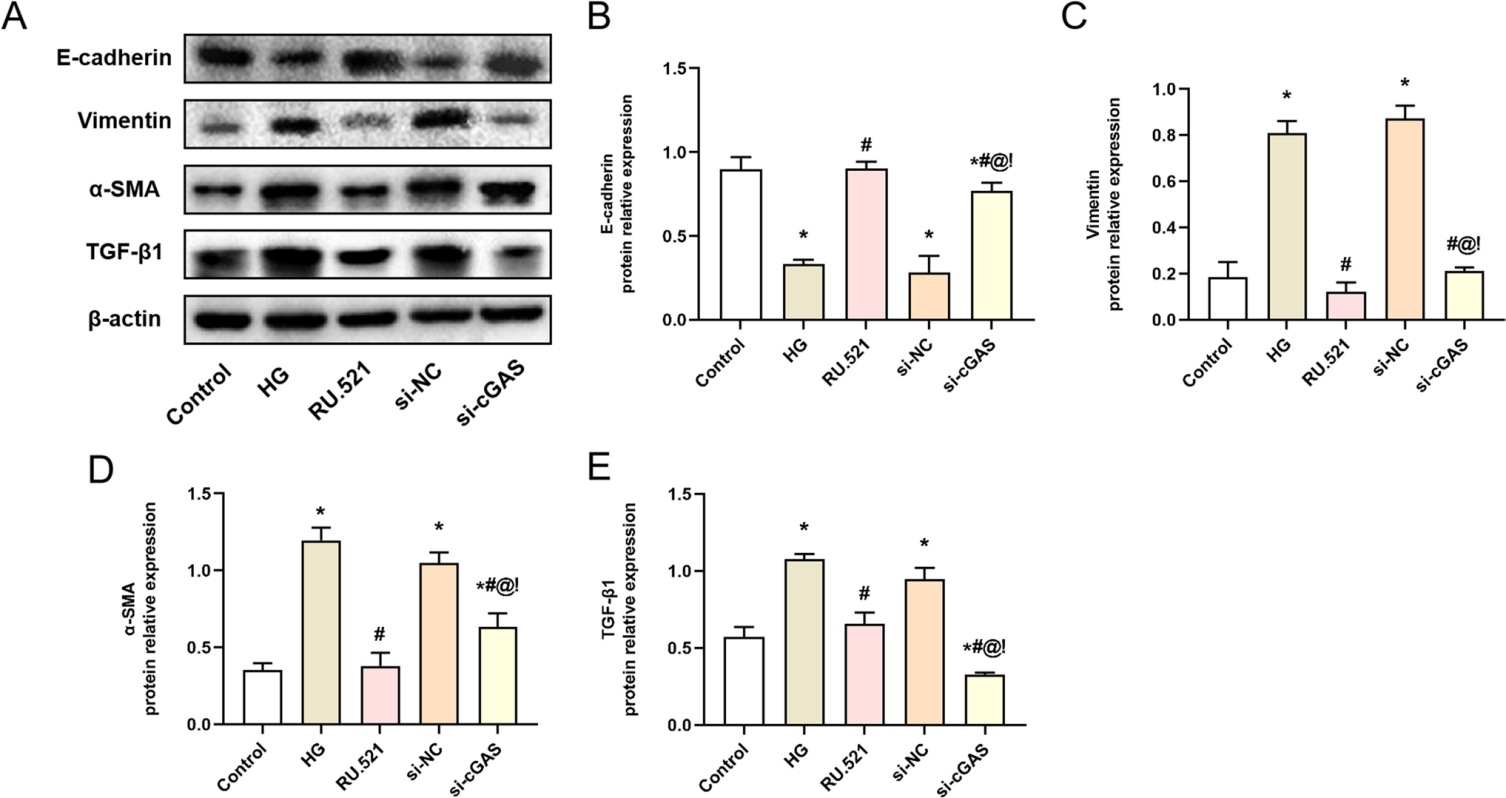
cGAS silencing alleviates the expression of MMT-related proteins induced by high glucose in HMrSV5 cells. (*A*) The test strips of WB. (*B*–*E*) Quantitative analysis of protein expression, (*B*) E-cadherin, (*C*) Vimentin, (*D*) α-SMA, (*E*) TGF-β1. Data are presented as the mean ± SD. *P < 0.05 vs. Control, #*P* < 0.05 vs. HG.

As shown in Fig. 6*A*, the MMT-related protein (E-cadherin, Vimentin, α-SMA, and TGF-β1) expressions in HMrSV5 cells were detected by WB. The analysis results are shown in Fig. 6*B*–*E*. Compared with the Control group, the Vimentin, α-SMA, and TGF-β1 protein expressions of HMrSV5 cells in the HG group were significantly increased (*P* < 0.05), while the E-cadherin protein expression of HMrSV5 cells in the HG group was significantly decreased (*P* < 0.05). However, compared with the HG group, the Vimentin, α-SMA, and TGF-β1 protein expressions of HMrSV5 cells in the RU.521 group and the si-cGAS group were significantly decreased (*P* < 0.05), while the E-cadherin protein expression of HMrSV5 cells was significantly increased (*P* < 0.05). These results indicate that interfering with the cGAS-STING signaling pathway can effectively suppress HG-induced MMT in HMrSV5 cells.

## Discussion

Peritoneal fibrosis (PF) represents a prevalent lesion type among peritoneal dialysis patients. The prolonged exposure to various adverse factors during long-term peritoneal dialysis treatment induces structural and functional damage to the peritoneum, ultimately resulting in PF (Bai *et al*. 2023; Stepanova *et al.* 2023; Zhao *et al*. 2023). EMT can be triggered under diverse pathological conditions, including hyperglycemia, hypoxia, and inflammation. During EMT, endothelial cells secrete a range of cytokines, such as TGF-β, VEGF, and TNF-α, which further facilitate the EMT and neovascularization of peritoneal mesothelial cells, thereby accelerating the progression of PF (Liu *et al*. 2015; Strippoli *et al*. 2016; Lu *et al.* 2023). In this study, we found that the expression of MMT-related proteins (α-SMA and vimentin) is significantly promoted in human peritoneal mesothelial cells HMrSV5 exposed to 4.25% high glucose. These findings are consistent with prior studies (Li *et al*. 2019; Mo *et al*. 2023).

cGAS is a cytosolic DNA sensor that not only detects pathogenic DNA from exogenous viruses and bacteria but also responds to endogenous DNA originating from mitochondria, retroelements, and chromosomal damage (Li *et al.* 2024; Wu *et al*. 2024). Upon activation by DNA, cGAS catalyzes the conversion of ATP and GTP into cGAMP, which subsequently binds to and activates the endoplasmic reticulum-resident protein STING. This interaction initiates a signaling cascade that leads to the activation of TBK1 and NF-κB pathways, thereby inducing the production of type I and type III interferons (IRF3), pro-inflammatory cytokines, chemokines, and interferon-stimulated gene expression products for the clearance of pathogens or damaged cells (Gong *et al*. 2024; Dimitrov *et al*. 2025). In this paper, we found that the cGAS, p-STING/STING, p-IRF3/IRF3, and p-TBK1/TBK1 protein expressions of HMrSV5 cells in the HG group were significantly increased compared with the Control group.

To further investigate the correlation between the activation of the cGAS-STING signaling pathway and the MMT in HG-induced peritoneal mesothelial cells, we employed two approaches: treatment with the cGAS inhibitor RU.521 and si-cGAS. Subsequently, we analyzed their effects on the MMT process in HMrSV5 cells. According to the results, compared with the HG group, the Vimentin, α-SMA, and TGF-β1 expressions of HMrSV5 cells in the RU.521 group and the si-cGAS group were significantly decreased, while the E-cadherin expression of HMrSV5 cells was significantly increased. These results suggest that inhibition of the cGAS-STING signaling axis may alleviate peritoneal fibrosis by reducing the MMT of HG-induced HMrSV5 cells. Interestingly, Jiao *et al*. found that inhibition of the cGAS-STING signaling pathway with the cGAS inhibitor RU.521 could inhibit the development of renal fibrosis in mice (Jiao *et al*. 2025). In addition, Han *et al*. demonstrated that intracranial injection of RU.521 in a mouse model of hypertensive heart disease effectively suppressed myocardial fibrosis levels in mice (Han *et al*. 2022). Combined with the results of this study, RU.521 may serve as a promising small-molecule therapeutic agent for the treatment of PF. Interestingly, the cGAS-STING signaling pathway not only is implicated in the progression of PF but also plays a role in the development of various kidney diseases. Jiang *et al*. demonstrated that under hypoxic conditions, activation of the cGAS-STING signaling pathway exacerbates hypoxia-reoxygenation-induced renal fibrosis in mice (Jiang *et al*. 2023). Luo *et al*. reported that the cGAS-STING pathway is activated in a lipopolysaccharide (LPS)-induced mouse model of sepsis-associated acute kidney injury, and administration of the cGAS inhibitor RU.521 or the STING inhibitor DMXAA can alleviate renal damage in this model (Luo *et al*. 2024). Zang *et al*. found that the cGAS-STING signaling pathway participates in the regulation of podocyte injury in diabetic nephropathy (Zang *et al*. 2022).

In summary, the results of this study showed that suppression of the cGAS-STING signaling pathway mitigates HG-induced MMT in the human peritoneal mesothelial cell line HMrSV5. The main novelty of this study lies in the first exploration of the role of the cGAS-STING signaling pathway in the MMT of peritoneal mesothelial cells HMrSV5. Besides, through dual validation using siRNA interference (si-cGAS) and a cGAS inhibitor (RU.521), we confirmed that high glucose promotes MMT in these cells by activating the cGAS-STING signaling pathway. However, this study also has some limitations. Additional animal and clinical investigations are necessary to obtain conclusive evidence regarding the efficacy of RU.521 and its regulatory impact on PF. In addition, it remains unclear how elevated glucose levels induce activation of the cGAS-STING signaling pathway in HMrSV5 cells. Previous studies have demonstrated that a high-glucose environment promotes excessive mitochondrial ROS production, which can lead to mitochondrial dysfunction and subsequent leakage of mtDNA into the cytoplasm. Once released, mtDNA acts as a damage-associated molecular pattern that is recognized by cGAS, thereby initiating downstream signaling (Xiong *et al*. 2023; Wang *et al*. 2024a, b). We will further investigate its underlying mechanisms in our subsequent research.

## Supplementary Information

Below is the link to the electronic supplementary material.

Supplementary file 1 (PDF 1.14 MB)

Supplementary file 2 (PDF 134 KB)

## Data Availability

All data of this article are available on request from the corresponding author.
