# Supplementary material for: Inhibition of cGAS-STING signaling pathway alleviates high glucose-induced mesothelial-mesenchymal transition in human peritoneal mesothelial cell line HMrSV5
Source: In Vitro Cell Dev Biol Anim. 2025 Aug 28;61(9):1097–106. doi: 10.1007/s11626-025-01107-1 (PMC12628494; doi:10.1007/s11626-025-01107-1)

细胞 STR 检测报告

一、样品名称/编号：HMrSV5,BNCC358140

二、检测项目：STR 基因型检验

三、检测方法：用天根的基因组抽提试剂盒提取 DNA，采用 21-STR 扩增方案扩增，在 Seqstudio 型遗传分析仪上对 STR 位点和性别基因 Amelogenin 进行检测。

四、检验结果：

1.检验基本情况

多等位基因	匹配细胞系	细胞库	EV 值	匹配说明
无	-	ExPASy	-	无匹配

多等位基因指三等位及以上基因现象。

本次检测各细胞分型结果良好。

2.样本描述

该株细胞 DNA 分型在细胞系检索中没有找到匹配的细胞系。本次检测在该细胞系中没有发现多等位基因。

匹配说明

待测细胞系与收录于 ATCC, DSMZ, JCRB 和 ExPASy 数据库的细胞系 STR 数据进行比对，未收录于以上细胞库的细胞系将无法匹配。

附表：HMrSV5 细胞的 STR 位点和 Amelogenin 位点的基因分型结果

HMrSV5 细胞			
Marker	Allele 1	Allele 2	Allele 3
D3S1358	15	15	——
vWA	14	15	——
D7S820	8	11	——
CSF1PO	12	12	——
Penta E	15	23	——
D8S1179	12	14	——
D21S11	29	31	——
D16S539	11	12	——
D2S1338	19	25	——
Penta D	10	14	——
D19S433	14.2	15.2	——
TH01	6	6	——
D13S317	8	11	——
TPOX	8	11	——
D18S51	12	12	——
D6S1043	12	13	——
AMEL	X	Y	——
D1S1656	15	16	——
D5S818	11	11	——
D12S391	19	20	——
FGA	23	23	——

附图：ExPASy 数据库比对结果

NA	Query	NA	NA	x,y	12	19,25	15	11	8,11	12,14	8,11	11,12	12	14.2,15.2	29,31	23	10,14	15,23	6	8,11	14,15
CVCL_3161	SCCH-196	8	78.57%	X	12			10,11	8,11		8,11	11,12							8,9	8,11	14,16
CVCL_E066	633	8	76.92%	X	10,12			11	8,11		11	11,12							8,9.3	8,11	14
CVCL_JK07	A549 EML4-ALK	8	76.92%	X	10,12			11	8,11		11	11,12							8,9.3	8,11	14
CVCL_VR66	A549 EML4-ALK-Luc2	8	76.92%	X	10,12			11	8,11		11	11,12							8,9.3	8,11	14

分型方案及位点分布

	方案 1	方案 2	方案 3	方案 4
1	D3S1358	D8S1179	D19S433	AMEL
2	vWA	D21S11	TH01	D1S1656
3	D7S820	D16S539	D13S317	D5S818
4	CSF1PO	D2S1338	TPOX	D12S391
5	Penta E	Penta D	D18S51	FGA
6			D6S1043	
7				

检测人：张震

审核人：殷世腾

签发日期：2025.05.16

河南省工业微生物菌种工程技术研究中心

Henan Engineering Research Center of Industrial Microbiology

网址：www.bncc.com 电话：400-6699-833

Sample File	Sample Name	Panel	SQO	OS	SQ
55_G07_Cellidentification-0515pi-1-0516.fsa	HMrSV5	21Plex_STR_Panel_v1.2			

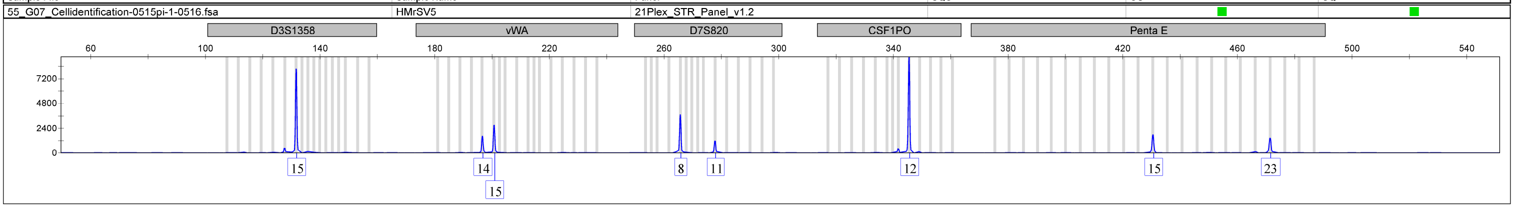

Sample File	Sample Name	Panel	SQO	OS	SQ
55_G07_Cellidentification-0515pi-1-0516.fsa	HMrSV5	21Plex_STR_Panel_v1.2			

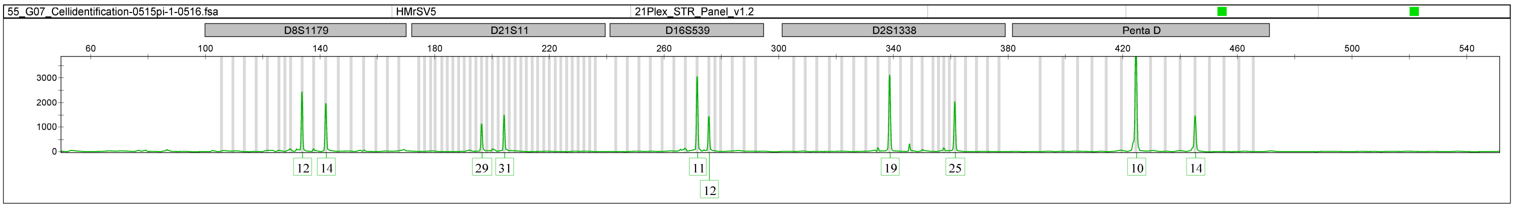

Sample File	Sample Name	Panel	SQO	OS	SQ
55_G07_Cellidentification-0515pi-1-0516.fsa	HMrSV5	21Plex_STR_Panel_v1.2			

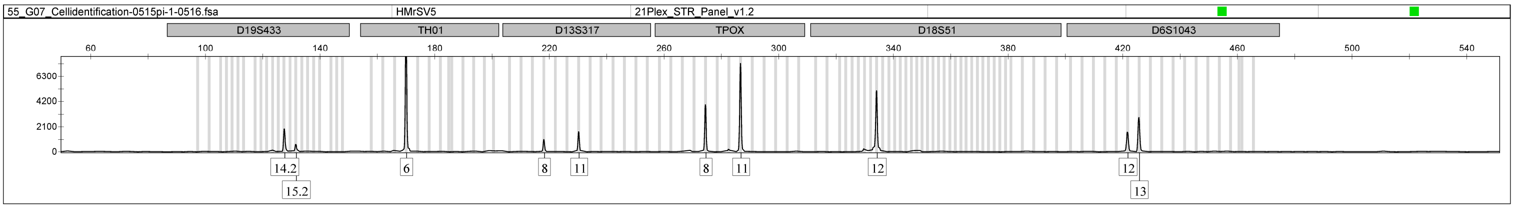

Sample File	Sample Name	Panel	SQO	OS	SQ
55_G07_Cellidentification-0515pi-1-0516.fsa	HMrSV5	21Plex_STR_Panel_v1.2			

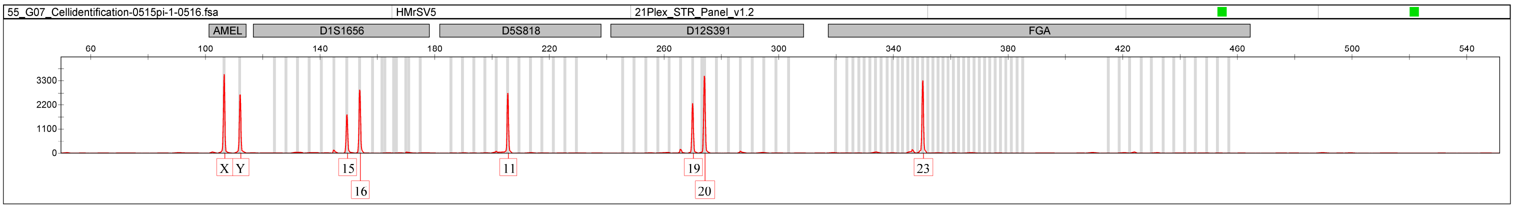

Supplement: Supplementary file 1 — (PDF 1.14 MB) [file 11626_2025_1107_MOESM1_ESM.pdf]
