# Supplementary material for: Inhibition of cGAS-STING signaling pathway alleviates high glucose-induced mesothelial-mesenchymal transition in human peritoneal mesothelial cell line HMrSV5
Source: In Vitro Cell Dev Biol Anim. 2025 Aug 28;61(9):1097–106. doi: 10.1007/s11626-025-01107-1 (PMC12628494; doi:10.1007/s11626-025-01107-1)
Supplement: Supplementary file 2 — (PDF 134 KB) [file 11626_2025_1107_MOESM2_ESM.pdf]

细胞编号	BNCC358140	检测日期	2024/8/14
细胞名称	HMrSV5 人腹膜间皮细胞	打印日期：	2024/8/14
检测项目	细胞支原体检测	检测人：	全文涵
检测方法	实时荧光定量 PCR 法	审核人：	何立胜

<table>
<tr><td rowspan="3">CT值</td><td colspan="2">通道</td><td>CT值</td></tr>
<tr><td colspan="2">FAM</td><td>N/A</td></tr>
<tr><td colspan="2">ROX</td><td>28.48</td></tr>
<tr><td>曲线图</td><td colspan="3">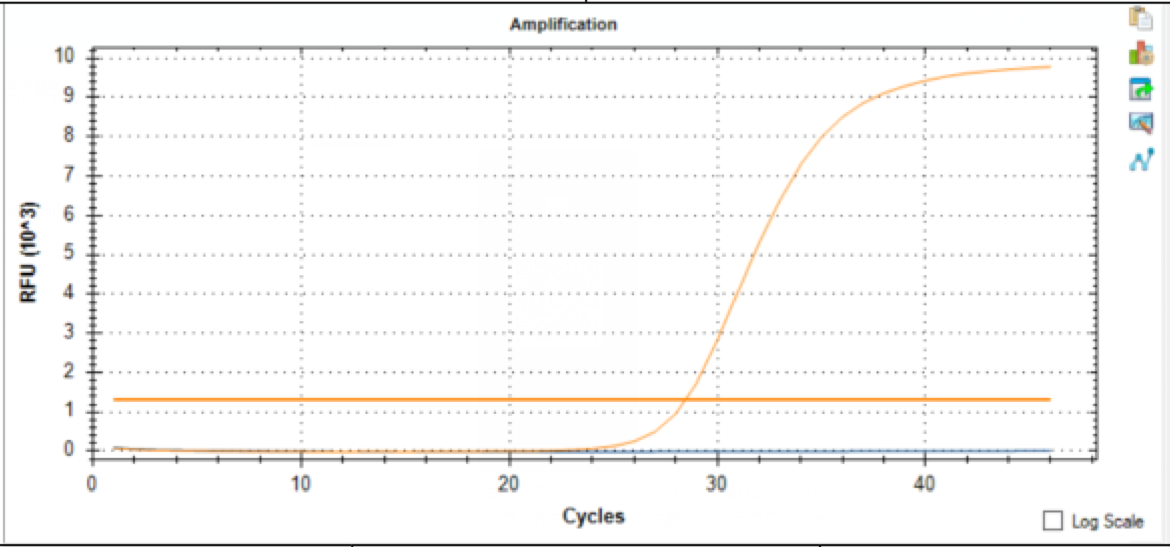</td></tr>
<tr><td rowspan="6">判定依据</td><td>FAM Channel</td><td>ROX Channel</td><td>Interpretation</td></tr>
<tr><td>Positive control</td><td>Ct<35</td><td>Reagents works</td></tr>
<tr><td>Positive control</td><td>Ct>35</td><td>Reagents not work</td></tr>
<tr><td>Sample no Ct</td><td>Ct<35</td><td>Negative</td></tr>
<tr><td>Sample Ct<40</td><td>Ct<35</td><td>Positive</td></tr>
<tr><td>Sample no Ct</td><td>Ct>35</td><td>PCR inhibition</td></tr>
<tr><td>检测结论</td><td colspan="3">细胞未受到支原体污染</td></tr>
</table>
